# Portrait of the journal as a young adult

**DOI:** 10.1186/1476-069X-11-30

**Published:** 2012-05-02

**Authors:** Philippe Grandjean, David Ozonoff

**Affiliations:** 1 Department of Environmental Medicine, University of Southern Denmark, 5000, Odense, Denmark; 2 Department of Environmental Health, Harvard School of Public Health, Boston, MA, USA; 3 Department of Environmental Health, Boston University School of Public Health, Boston, MA, USA

**Keywords:** Access numbers, Decision making, Environmental health science, Impact factor, Least publishable unit, Publishing, Publication bias

## Abstract

Since its founding a decade ago, *Environmental Health* has received more than one thousand manuscripts. Last year marked the first time we published over 100 articles. The journal web site currently receives over 60,000 unique visitors every month, and the steady increase suggests that the number will soon exceed 100,000 monthly. Individual articles are usually accessed several thousand times within a few years of publication, the highest number for a single paper being close to 100,000. The access numbers suggest that the journal has a reach that goes much beyond narrow academic circles as represented by numbers of citations in scholarly journals. To further the goal of reaching the wider public health community and linking to environmental health promotion, we ask prospective *Environmental Health* authors to highlight the findings that are new or different along with the implications for future research and in terms of prevention of environmental hazards.

## Status after ten years

*Environmental Health* recently passed two significant milestones, the end of its first decade and the first time the journal has published more than 100 papers in a single year (109). During this span we received 1,000 manuscripts for consideration, 248 submissions alone in our tenth year. Road markers like this provide a good opportunity for stocktaking.

*Environmental Health* is an internet-based journal, potentially allowing faster processing of manuscripts. It is our intention to put papers through the editorial process as rapidly as possible, consistent with time for constructive peer review. We do not consider peer review a gate-keeping function but a collegial one. Our Open Review process, where both the reviewers and authors are identified to each other, emphasizes this philosophy [1]. We take ‘peer review’ literally – we mean it to be the constructive and friendly review you would expect to get from a colleague you asked to read your paper and provide you with feedback. Sometimes that feedback indicates that the paper is not ready for publication because of gaps, problems or insufficient substance. Much more often, the feedback consists in requests for clarification or constructive suggestions on how to make the paper clearer, more meaningful and more persuasive in its conclusions. We consider this the greatest ‘value added’ of our publication process and we hope that you do, too.

This kind of collegial consultation sometimes takes time, however, as the most competent reviewers usually are the busiest. We are extremely grateful to our reviewers, but we are also at their mercy. Producing a reliable and quality product takes time, and while we are swifter than our print counterparts, some things can’t be hurried too much and peer review is an area where we don’t cut corners. The whole process, from date of submission to publication, averages just above five months. Once a manuscript has been approved, publication is rapid. The online format means that we are not constrained by print space but can publish any article deemed worthy by reviewers and the Editors and, importantly, that falls within the *Environmental Health* interests of our international readership.

## Acceptance rates and least publishable units

Because we work with authors and reviewers to improve manuscripts (thereby extending the average time to publication), our overall acceptance rate is close to 50 % overall (910 decisions by the end of 2011, with 449 acceptances). An important reason for not accepting papers relates to appropriateness of the subject matter (to be discussed below). We publish papers of various lengths and degrees of specificity, but it would not be completely honest to say we are always satisfied with all of the manuscripts that we publish, not for lack of rigor or uninteresting subjects, but because we often receive fragments of work that the authors hope will meet the threshold of the Least Publishable Unit (LPU).

We understand there are many reasons for submitting an LPU, not the least of which are institutional and funding agency demands for tangible products. But there is a downside, too. It requires scientists to examine multiple publications to piece together the findings of a particular study. Larger studies divided into short papers provide only a partial view of what was seen and are more likely to suffer from citation amnesia [2]. Pilot studies and preliminary reports are important in the research process, but this importance does not necessarily imply that they should be published in a scholarly journal. A pilot study might be so interesting, novel or otherwise significant that it deserves a stand-alone publication. But it will have to make the case. The journal will continue to select quality and overall significance over quantity or incremental advance.

## Journal focus

We continue to focus on the human health aspects of environmental hazards. This means that we will in general not process submissions on laboratory models of unclear validity or other studies of limited relevance to human health, no matter how strong or significant the effects of the purported toxicant or other hazard. We look for a clear link and immediate relevance to human health. Toxicology is extremely important to our discipline, but purely toxicological papers are for other journals than ours. There will, of course, be the occasional exception due to novelty and importance to the field in general. However, we have become concerned about the problematic tendency of covering repeatedly the same territory.

We recently published an article that examined the chemicals that had been covered by (other) Environmental Health journals during the first ten years of this millennium [3]. This journal was not included, as we did not yet have ten years of publication records. Metals – notably lead, cadmium, nickel, chromium, arsenic, and mercury – were immensely popular in the 78 journals covered by the study, with between 4,000 and 10,000 articles on each of the most popular elements during the ten years. Polychlorinated biphenyls, solvents, and polyaromatic hydrocarbons were also common subjects, but substances like perfluorinated alkane compounds, bisphenol A, and triclosan were much less so. The comparable data for *Environmental Health*, which we have now collated, is not much different, unfortunately. Air pollutants are popular topics in our journal, and we have published 14 articles on lead, 13 on the pesticide metabolite DDE, and five on dioxin. Although there is probably much yet to be learned about these well-researched environmental chemicals, the statistic raises a concern whether we focus enough on new environmental hazards that we ought to examine in greater depth. Thus, it is disconcerting that substances identified by the U.S. Environmental Protection Agency as high priority in regard to environmental fate and toxicology are being virtually ignored in science journal publications [3].

As journal editors, we have little influence on our colleagues’ choice of research topics. But we would like to encourage attention to new or emerging problems in *Environmental Health*. Much of the conservatism or inertia is probably structural. Funding is sensitive to ‘track records’ and track records are tied to experience with particular chemicals. Success in the grant world is not correlated with broad horizontal strategies but with vertical or ‘drill down’ ones. This focus is reproduced in graduate students and may, possibly, also affect the next generation of graduate students. It is not just that deviating from the beaten path is conceptually difficult. The main obstacle is that novelty may be professionally hazardous. Our journal will aim at decreasing any obstacles in this regard.

## Impact factor or a factor in impact?

Some of the chemicals most prominent in environmental science journals also benefit from high citation numbers [3] – possibly a self-propagating mechanism, where frequent publication within a specialized area generates more citations and then again more publications. Journal editors will of course aim for the highest possible impact factor, as determined by recent citations in other articles. However, our primary goal for *Environmental Health* is scientific and social impact, rather than citation records. Thus, we hope that the authors submitting to *Environmental Health* will generate the innovation and inspiration that we and our colleagues in the field desire.

**Figure 1:**
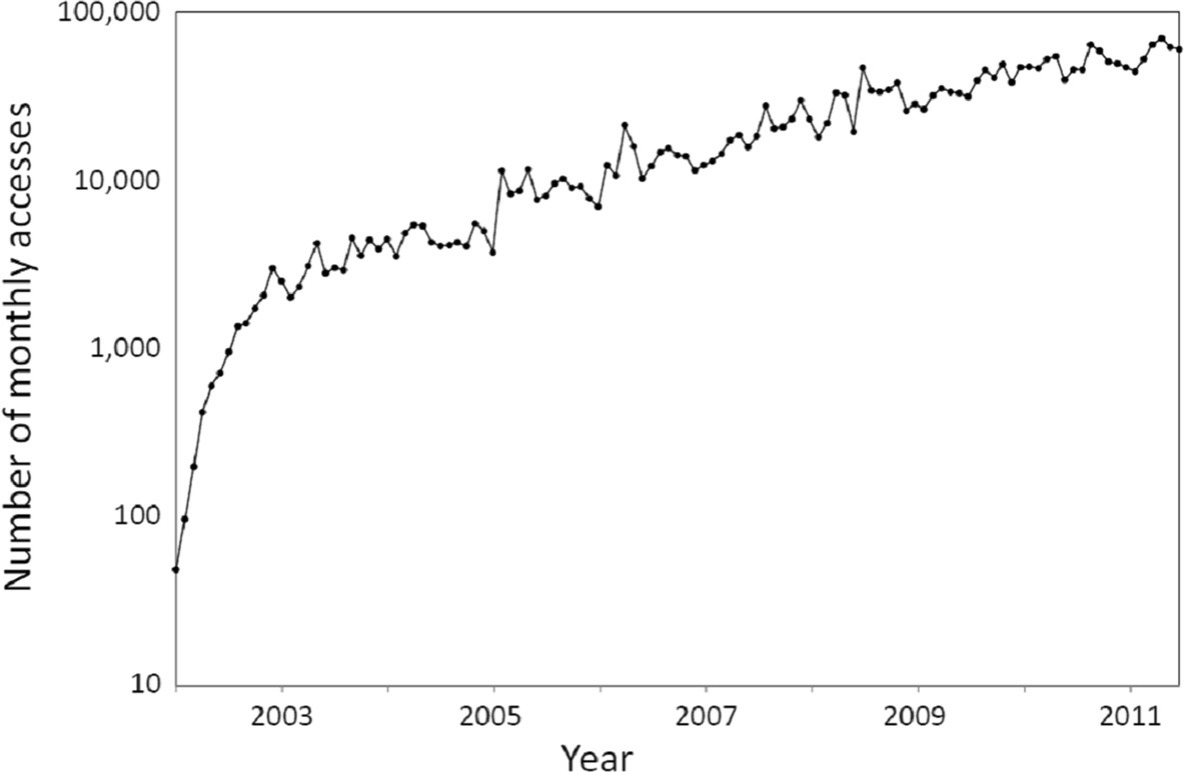
**Total number of monthly article accesses (logarithmic scale) at the** *** Environmental Health* web site between July 2002 and February 2012.**

The statistics from the *Environmental Health* web site suggest that our journal is attracting attention. Our website has about 1,000 unique visits every weekday. The number of monthly accesses has steadily grown to more than 60,000, and hopefully we will reach 100,000 by next year (Figure 1). The users are from over 150 countries, with the United States, the United Kingdom, Canada, and India among the most prevalent. PubMed is our most important referral site, and Google the most important search engine. PubMed also allows readers to access published *Environmental Health* articles through the repository at PubMed Central. Since we have no data on accesses via this source, our counts are minimum numbers. Considering that we started out with 49 accesses in July of 2002 when we published the first article, these numbers show that *Environmental Health* has become well established as a global information source.

**Figure 2:**
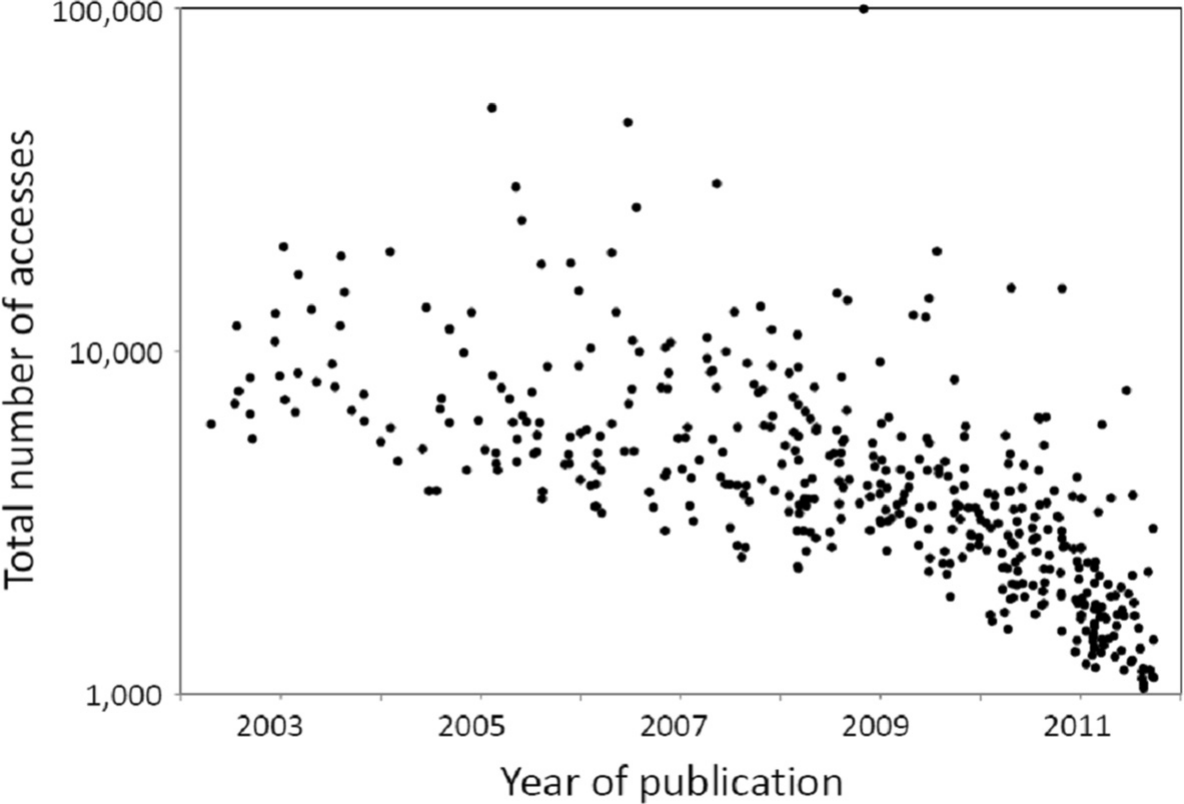
**Number of accesses (logarithmic scale) at the * Environmental Health* web site for individual articles in relation to the time of publication.**

The most popular *Environmental Health* article has been accessed so far close to 100,000 times within three years of its publication, while the runner-up has been accessed almost 50,000 times. More than 40 articles (about 10% of all published) have been accessed over 10,000 times. More than half of these articles were published in 2007 or earlier, as access numbers accumulate with time (Figure 2). Still, even the most specialized articles are routinely accessed several hundred times during the first month or two of publication. Authors can follow the access records of their own manuscripts, and readers can retrieve overall rankings of articles. These options are unique to journals like ours, and are popular among our colleagues.

**Figure 3:**
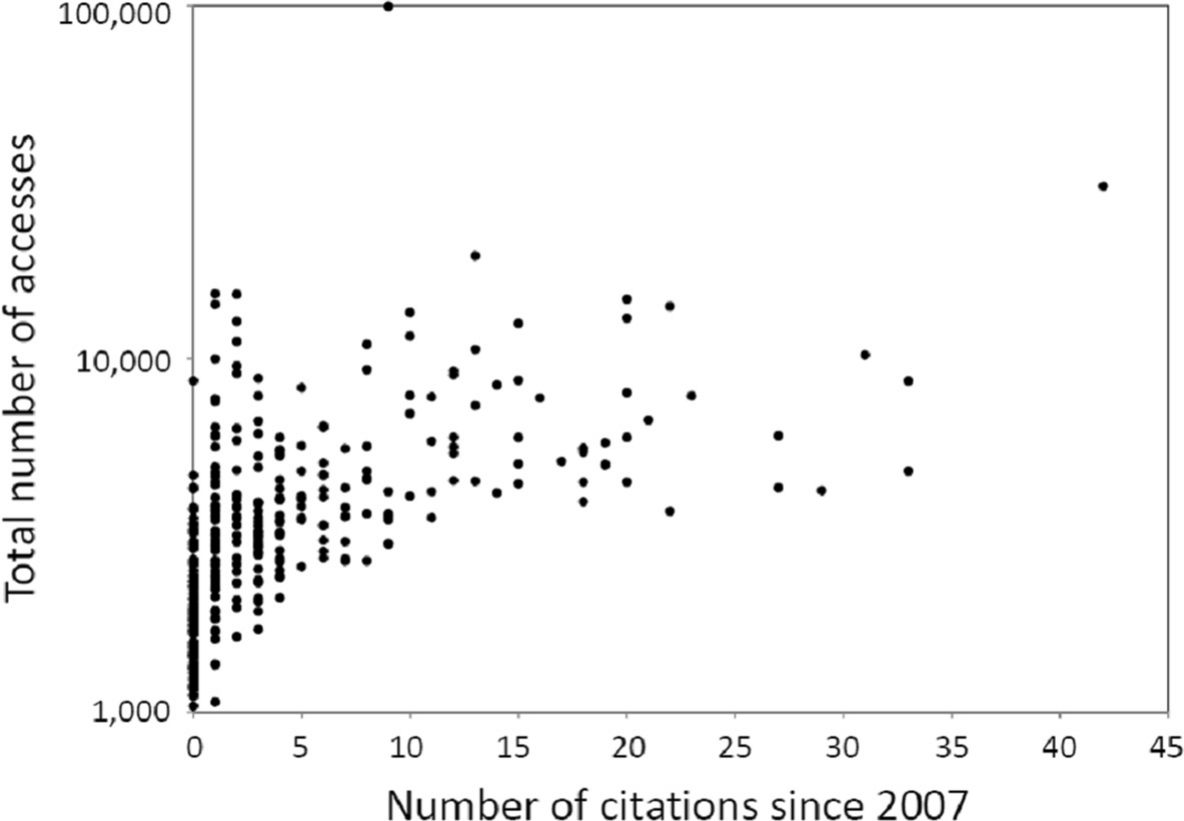
**Number of accesses (logarithmic scale) at the** *** Environmental Health* web site for individual articles published since 2007 in relation to their numbers of citations at the Web of Science through March, 2012.**

There is an important difference between attracting attention from the readership and being cited in scientific journals. Several articles accessed at our web site at least 10,000 times have just been cited once or twice since 2007 (when the Institute for Scientific Information started tracking our publications). Nevertheless, Figure 3 shows a clear, positive correlation between numbers of citations and accesses. Those not cited yet or cited up to four times had been accessed on average less than 4,000 times, while articles cited at least five times had been accessed over 7,000 times on average. The highest number of citations (42) was an *Environmental Health* article accessed more than 30,000 times by March, 2012. The article accessed 100,000 times has accumulated nine citations, while being widely referred to at internet sources not included in the citation records (citations listed are from the Web of Science, but citation numbers of *Environmental Health* articles are about 10-20% higher at the Scopus database, which covers citations in a wider range of scientific publications). We believe this is another indication that papers about novel topics may achieve a high profile and visibility but not be cited because there are far fewer investigators writing on the topic in scholarly journals.

Our impact factor remained at a very respectable level in 2011, placing our journal in the top quartile. And we believe it will increase this year and beyond. Before the internet, specialty journals often had to be content with subscriptions below 1000. Our download statistics document that free research information in our field has become rapidly and widely available. Still the numbers suggest that our journal is a factor in overall impact beyond the statistic called the Impact Factor.

Download numbers and impact factors are really reflections of a much bigger issue – the role of a journal such as ours. The question is what kind of an impact in a wider sense that a journal such as ours should have. There is clearly much at stake in a changing world, where serious environmental hazards abound. The very purpose of research should be to provide new information, new insight, and perhaps to upset our current knowledge. As Bradford Hill famously wrote almost 50 years ago [4], “All scientific work is incomplete… All scientific work is liable to be upset or modified by advancing knowledge”.

Research is an incremental process. Some subjects are not yet amenable to full-scale epidemiological scrutiny. Thus, a brief manuscript that appears like an LPU may herald an important new insight. One of us once declared that a workable definition of a public health catastrophe was a health effect so powerful even an epidemiological study could detect it. It was made in jest but contains a bitter truth: problems that are difficult to specify may still develop to alarming proportions without being amenable to epidemiological research.

## Moving science forward

*Environmental Health* research includes elements of both basic and applied science. Its implications rely on a high quality and relevance. The translational aspects of the research are oftentimes downplayed, as researchers do not want to cry ‘wolf’ too often and want to avoid being looked upon as advocates for particular policies or interventions. But hedging over-cautious conclusions and hiding in the ivory tower will have social costs that will inevitably also hurt environmental health research if it is perceived as a science too removed from real-world problems.

In addition to the traditional evaluation of whether the results could have arisen by chance given no bias or real effect, we also need to determine how large an effect could be consistent with the results observed. Both extremes of the confidence intervals are related to the stability of the point estimate and should be expressed by information beyond a p-value. We therefore ask that results be presented with estimates of the stability of the results with an appropriately stated and calculated confidence interval.

We need to get away from rigid markers when interpreting data. There are many instances when results that are very statistically significant but of no or little public health significance. Conversely, there are many instances when observations that do not reach the level of statistical significance signal concerns of utmost public health significance. Bradford Hill also said that incomplete evidence “does not confer upon us the freedom to ignore the knowledge we already have, or to postpone the action that it appears to demand at the given time” [4].

Statistical requirements are only one aspect of the worth of a scientific result. In a recent Commentary in *Nature*, Jerome Ravetz makes a key observation: ultimately, a necessary condition for good quality is trust [5]. When we read a paper we invest its author with an extraordinary amount of confidence: that the citations say what the author alleges they say (although we can check this, few people check every reference; we take it on trust); that the methods were conducted as described; that the results accurately reflected what was actually found, etc. Trust is earned through mutual respect, civility and a reputation for intellectual honesty. As Editors of a scientific journal we hope to do our part to foster these virtues through the review process and through our editorial evaluations and decisions. We also rely on our readers through our open peer review, where reviews and author responses are made accessible to the readers.

One additional requirement follows from this. Articles published in *Environmental Health* should contain enough information – or raw data submitted in supplementary files – to allow a subsequent meta-analysis or replication. We recognize that making data publicly available can also be problematic [6], but we ask our authors to consider favorably the possibility.

Decision-making on environmental protection is complex and often seems to be lagging behind the scientific insights, most obviously in regard to climate change. But noise, air pollution, and various food contaminants are also good examples. While we recognize the complexities in achieving equitable and affordable prevention, we believe that science should provide its best possible input to identification of responsible decisions. We do not advance public health and environmental protection by concluding with the truism that ‘further research is needed.’ We are proud of our successful outreach, but it entails added responsibilities in regard to appropriate scientific inference. We ask our authors to share that responsibility with us. If they forget or overlook this obligation, we shall be sure to remind them. And if we somehow fail, we ask our readers to provide the missing angle by using our ‘Comment’ function. We look forward to hearing from you as authors, reviewers, commentators, or in any other capacity that can help us strengthen our journal and advance *Environmental Health* science.

## Abbreviation

LPU: Least Publishable Unit.

## Competing interests

PG and DO are founding editors-in-chief of *Environmental Health*.

## Authors’ contributions

PG drafted the first version of the manuscript, and both authors contributed to and approved the final version. Both authors read and approved the final manuscript.
